# Conclusions of the Worldwide Integrated Assessment on the risks of neonicotinoids and fipronil to biodiversity and ecosystem functioning

**DOI:** 10.1007/s11356-014-3229-5

**Published:** 2014-10-10

**Authors:** J. P. van der Sluijs, V. Amaral-Rogers, L. P. Belzunces, M. F. I. J. Bijleveld van Lexmond, J-M. Bonmatin, M. Chagnon, C. A. Downs, L. Furlan, D. W. Gibbons, C. Giorio, V. Girolami, D. Goulson, D. P. Kreutzweiser, C. Krupke, M. Liess, E. Long, M. McField, P. Mineau, E. A. D. Mitchell, C. A. Morrissey, D. A. Noome, L. Pisa, J. Settele, N. Simon-Delso, J. D. Stark, A. Tapparo, H. Van Dyck, J. van Praagh, P. R. Whitehorn, M. Wiemers

**Affiliations:** 1 Department of Environmental Sciences, Copernicus Institute, Utrecht University, Heidelberglaan 2, 3584 CS Utrecht, The Netherlands; 2 Buglife, Bug House, Ham Lane, Orton Waterville, Peterborough, PE2 5UU UK; 3 INRA, UR 406 Abeilles and Environnement, Laboratoire de Toxicologie Environnementale, Site Agroparc, 84000 Avignon, France; 4 46 Pertuis-du-Sault, 2000 Neuchâtel, Switzerland; 5 Centre National de la Recherche Scientifique, Centre de Biophysique Moléculaire, Rue Charles Sadron, 45071 Orléans Cedex 02, France; 6 Département des Sciences Biologiques, Université du Québec À Montréal, Case Postale 8888, Succursale Centre-Ville, Montreal, Québec Canada H3C 3P8; 7 Haereticus Environmental Laboratory, P.O. Box 92, Clifford, VA 24533 USA; 8 Veneto Agricoltura, Legnaro, PD Italy; 9 RSPB Centre for Conservation Science, RSPB, The Lodge, Sandy, Bedfordshire SG19 2DL UK; 10 Department of Chemistry, University of Cambridge, Lensfield Road, CB2 1EW Cambridge, UK; 11 Dipartimento di Agronomia Animali Alimenti Risorse Naturali e Ambiente, Università degli Studi di Padova, Agripolis, viale dell’Università 16, 35020 Legnaro, Padova Italy; 12 School of Life Sciences, University of Sussex, Brighton, BN1 9RH UK; 13 Canadian Forest Service, Natural Resources Canada, 1219 Queen Street East, Sault Ste Marie, ON Canada P6A 2E5; 14 Department of Entomology, Purdue University, West Lafayette, IN 47907-2089 USA; 15 Department of System Ecotoxicology, Helmholtz Centre for Environmental Research - UFZ, 04318 Leipzig, Germany; 16 Healthy Reefs for Healthy People Initiative, Smithsonian Institution, Belize City, Belize; 17 Pierre Mineau Consulting, 124 Creekside Drive, Salt Spring Island, V8K 2E4 Canada; 18 Laboratory of Soil Biology, University of Neuchatel, Rue Emile Argand 11, 2000 Neuchatel, Switzerland; 19 Jardin Botanique de Neuchâtel, Chemin du Perthuis-du-Sault 58, 2000 Neuchâtel, Switzerland; 20 Department of Biology and School of Environment and Sustainability, University of Saskatchewan, 112 Science Place, Saskatoon, SK S7N 5E2 Canada; 21 Task Force on Systemic Pesticides, Pertuis-du-Sault, 2000 Neuchâtel, Switzerland; 22 Kijani, Kasungu National Park, Private Bag 151, Lilongwe, Malawi; 23 Department of Community Ecology, Helmholtz Centre for Environmental Research - UFZ, Theodor-Lieser-Str. 4, 06120 Halle, Germany; 24 iDiv, German Centre for Integrative Biodiversity Research, Halle-Jena-Leipzig, Deutscher Platz 5e, 04103 Leipzig, Germany; 25 Beekeeping Research and Information Centre (CARI), Place Croix du Sud 4, 1348 Louvain la Neuve, Belgium; 26 Puyallup Research and Extension Centre, Washington State University, Puyallup, WA 98371 USA; 27 Dipartimento di Scienze Chimiche, Università degli Studi di Padova, via Marzolo 1, 35131 Padova, Italy; 28 Behavioural Ecology and Conservation Group, Biodiversity Research Centre, Université Catholique de Louvain (UCL), Croix du Sud 4-5 bte L7.07.04, 1348 Louvain-la-Neuve, Belgium; 29 Scientific Advisor, Hassellstr. 23, 29223 Celle, Germany; 30 School of Natural Sciences, University of Stirling, Stirling, FK9 4LA UK; 31 Centre for the Study of the Sciences and the Humanities, University of Bergen, Postboks 7805, N-5020 Bergen, Norway

## Introduction

The side effects of the current global use of pesticides on wildlife, particularly at higher levels of biological organization: populations, communities and ecosystems, are poorly understood (Köhler and Triebskorn 2013). Here, we focus on one of the problematic groups of agrochemicals, the systemic insecticides fipronil and those of the neonicotinoid family. The increasing global reliance on the partly prophylactic use of these persistent and potent neurotoxic systemic insecticides has raised concerns about their impacts on biodiversity, ecosystem functioning and ecosystem services provided by a wide range of affected species and environments. The present scale of use, combined with the properties of these compounds, has resulted in widespread contamination of agricultural soils, freshwater resources, wetlands, non-target vegetation and estuarine and coastal marine systems, which means that many organisms inhabiting these habitats are being repeatedly and chronically exposed to effective concentrations of these insecticides.

Neonicotinoids and fipronil currently account for approximately one third (in monetary terms in 2010) of the world insecticide market (Simon-Delso et al. 2014). They are applied in many ways, including seed coating, bathing, foliar spray applications, soil drench applications and trunk injection. These compounds are used for insect pest management across hundreds of crops in agriculture, horticulture and forestry. They are also widely used to control insect pests and disease vectors of companion animals, livestock and aquaculture and for urban and household insect pest control and timber conservation (Simon-Delso et al. 2014).

Although the market authorization of these systemic insecticides did undergo routine ecological risk assessments, the regulatory framework has failed to assess the individual and joint ecological risks resulting from the widespread and simultaneous use of multiple products with multiple formulations and multiple modes of action. These applications co-occur across hundreds of cropping systems including all of our major agricultural commodities worldwide and on numerous cattle species, companion animals, etc. Also, the ecological risk assessment did not consider the various interactions with other environmental stressors. Once a market authorization is granted, the authorization poses limits to the dose and frequency per allowed application, but no limits are set to the total scale of use of the active ingredients leading to a reduced potential for the recovery of impacted ecosystems from effects. In addition, there has been no assessment of successive neonicotinoid exposure typical in watersheds and resulting in culmination of exposure and effects over time (Liess et al. 2013). The potential interactions between neonicotinoids and fipronil and other pesticide active substances have not been considered either, although additivity and synergisms of toxic mechanisms of action have been documented (Satchivi and Schmitzer 2011; Gewehr 2012; Iwasa et al. 2004).

The Worldwide Integrated Assessment (WIA) presented in the papers in this special issue is the first attempt to synthesize the state of knowledge on the risks to biodiversity and ecosystem functioning posed by the widespread global use of neonicotinoids and fipronil. The WIA is based on the results of over 800 peer-reviewed journal articles published over the past two decades. We assessed respectively the trends, uses, mode of action and metabolites (Simon-Delso et al. 2014); the environmental fate and exposure (Bonmatin et al. 2014); effects on non-target invertebrates (Pisa et al. 2014); direct and indirect effects on vertebrate wildlife (Gibbons et al. 2014); and risks to ecosystem functioning and services (Chagnon et al. 2014) and finally explored sustainable pest management practices that can serve as alternatives to the use of neonicotinoids and fipronil (Furlan and Kreutzweiser 2014).

## Mode of action, environmental fate and exposure

Due to their systemic nature, neonicotinoids and, to a lesser extent, fipronil as well as several of their toxic metabolites are taken up by the roots or leaves and translocated to all parts of the plant, which, in turn, makes the treated plant effectively toxic to insects that are known to have the potential to cause crop damage. Neonicotinoids and fipronil operate by disrupting neural transmission in the central nervous system of organisms. Neonicotinoids bind to the nicotinic acetylcholine receptor, whereas fipronil inhibits the GABA receptor. Both pesticides produce lethal and a wide range of sublethal adverse impacts on invertebrates but also some vertebrates (Simon-Delso et al. 2014 and Gibbons et al. 2014). Most notable is the very high affinity with which neonicotinoid insecticides agonistically bind to the nicotinic acetylcholine receptor (nAChR) such that even low-dose exposure over extended periods of time can culminate into substantial effects (see the literature reviewed by Pisa et al. 2014).

As a result of their extensive use, these substances are found in all environmental media including soil, water and air. Environmental contamination occurs via a number of disparate routes including dust generated during drilling of dressed seeds; contamination and build-up of environmental concentrations after repeated application in arable soils and soil water; run-off into surface and ground waters; uptake of pesticides by non-target plants via their roots followed by translocation to pollen, nectar, guttation fluids, etc.; dust and spray drift deposition on leaves; and wind- and animal-mediated dispersal of contaminated pollen and nectar from treated plants. Persistence in soils, waterways and non-target plants is variable but can be long; for example, the half-lives of neonicotinoids in soils can exceed 1,000 days. Similarly, they can persist in woody plants for periods exceeding 1 year. Breakdown results in toxic metabolites, though concentrations of these in the environment are rarely measured (Bonmatin et al. 2014).

This combination of persistence (over months or years) and solubility in water has led to large-scale contamination of, and the potential for build-up in, soils and sediments (ppb-ppm range), waterways (ground and surface waters in the ppt-ppb range) and treated and non-treated vegetation (ppb-ppm range). Screening of these matrices for pesticides and their metabolites has not been done in a systematic and appropriate way in order to identify both the long-term exposure to low concentrations and the short-term erratic exposure to high concentrations.

However, where environmental samples have been screened, they were commonly found to contain mixtures of pesticides, including neonicotinoids or fipronil (with their toxic metabolites). In addition, samples taken in ground and surface waters have been found to exceed limits based on regulatory ecological threshold values set in different countries in North America and Europe. Overall, there is strong evidence that soils, waterways and plants in agricultural and urban environments and draining areas are contaminated with highly variable environmental concentrations of mixtures of neonicotinoids or fipronil and their metabolites (Bonmatin et al. 2014).

This fate profile provides multiple routes for chronic and multiple acute exposure of non-target organisms. For example, pollinators (including bees) are exposed through at least direct contact with dust during drilling; consumption of pollen, nectar, guttation drops, extra-floral nectaries and honeydew from seed-treated crops; water; and consumption of contaminated pollen and nectar from wild flowers and trees growing near treated crops or contaminated water bodies. Studies of food stores in honeybee colonies from a range of environments worldwide demonstrate that colonies are routinely and chronically exposed to neonicotinoids, fipronil and their metabolites (generally in the 1–100 ppb range), often in combination with other pesticides in which some are known to act synergistically with neonicotinoids. Other non-target organisms, particularly those inhabiting soils and aquatic habitats or herbivorous insects feeding on non-crop plants in farmland, will also inevitably be exposed, although exposure data are generally lacking for these groups (Bonmatin et al. 2014).

## Impacts on non-target organisms

Impacts of systemic pesticides on pollinators are of particular concern, as reflected by the large number of studies in this area. In bees, field-realistic exposures in controlled settings have been shown to adversely affect individual navigation, learning, food collection, longevity, resistance to disease and fecundity. For bumblebees, colony-level effects have been clearly demonstrated, with exposed colonies growing more slowly and producing significantly fewer queens (Whitehorn et al. 2012). Limited field studies with free-living bee colonies have largely been inconsistent and proved difficult to perform, often because control colonies invariably become contaminated with neonicotinoids, or there is a lack of replication in the study design, all of which demonstrates the challenges of conducting such a study in the natural environment (Maxim and Van der Sluijs 2013; Pisa et al. 2014).

Other invertebrate groups have received less attention. For almost all insects, the toxicity of these insecticides is very high including many species that are important in biological control of pests. The sensitivity to the toxic effect is less clear with non-insect species. For annelids such as earthworms, the LC_50_ is in the lower ppm range for many neonicotinoids (LOEC at 10 ppb). Crustaceans are generally less sensitive, although sensitivity is highly dependent on species and developmental stage. For example, blue crab megalopae are an order of magnitude more sensitive than juveniles.

At field-realistic environmental concentrations, neonicotinoids and fipronil can have negative effects on physiology and survival for a wide range of non-target invertebrates in terrestrial, aquatic, wetland, marine and benthic habitats (see the literature reviewed by Pisa et al. 2014). Effects are predominantly reported from laboratory toxicity testing, using a limited number of test species. Such tests typically examine only lethal effects over short time frames (i.e. 48 or 96 h tests), whereas ecologically relevant sublethal effects such as impairment of flight, navigation or foraging ability and growth are less frequently described. It has become clear that many of the tests use insensitive test species (e.g. *Daphnia magna*) and are not sufficiently long to represent chronic exposure and therefore lack environmental relevance. Laboratory testing to establish safe environmental concentration thresholds is hindered by the fact that most pesticide toxicity tests are based on older protocols. Although these systemic pesticide classes possess many novel characteristics, testing methodologies have remained largely unchanged, resulting in flawed conclusions on their ecological safety (Maxim and Van der Sluijs 2013). New and improved methodologies are needed to specifically address the unique toxicology profiles of chemicals, including their possible cumulative and delayed lethal and non-lethal effects for a variety of terrestrial, aquatic and marine organisms. Nevertheless, our review shows a growing body of published evidence that these systemic insecticides pose a serious risk of harm to a broad range of non-target invertebrate taxa often below the expected environmental concentrations. As a result, an impact on the many food chains they support is expected.

We reviewed nearly 150 studies of the direct (toxic) and indirect (e.g. food chain) effects of fipronil and the neonicotinoids imidacloprid and clothianidin on vertebrate wildlife—mammals, birds, fish, amphibians and reptiles. Overall, at concentrations relevant to field exposure scenarios in fields sown with coated seeds, imidacloprid and clothianidin pose risks to small birds, and ingestion of even a few treated seeds could cause mortality or reproductive impairment to sensitive bird species (see the studies reviewed by Gibbons et al. 2014). Some recorded environmental concentrations of fipronil have been sufficiently high to potentially harm fish (Gibbons et al. 2014). All three insecticides exert sublethal effects, ranging from genotoxic and cytotoxic effects to impaired immune function, reduced growth or reduced reproductive success. Conclusive evidence was described recently, that neonicotinoids impair the immune response at the molecular level, thus enabling damages by covert diseases and parasites (Di Prisco et al. 2013). All these effects often occur at concentrations well below those associated with direct mortality (Gibbons et al. 2014). This is a trend in many taxa reported throughout the reviewed literature: short-term survival is not a relevant predictor neither of mortality measured over the long term nor of an impairment of ecosystem functions and services performed by the impacted organisms.

With the exception of the most extreme cases, the concentrations of imidacloprid and clothianidin that fish and amphibians are exposed to appear to be substantially below thresholds to cause mortality, although sublethal effects have not been sufficiently studied. Despite the lack of research and the difficulty in assigning causation, indirect effects may be as important as direct toxic effects on vertebrates and possibly more important. Neonicotinoids and fipronil are substantially more effective at killing the invertebrate prey of vertebrates than the vertebrates themselves. Indirect effects are rarely considered in risk assessment processes, and there is a paucity of data, despite the potential to exert population-level effects. Two field case studies with reported indirect effects were found in the published literature. In one, reductions in invertebrate prey from both imidacloprid and fipronil uses led to impaired growth in a fish species, and in another, reductions in populations of two lizard species were linked to effects of fipronil on termite prey (see the studies reviewed by Gibbons et al. 2014).

## Impacts on ecosystem functioning and ecosystem services

The concept of ecosystem services is widely used in decision-making in the context of valuing the service potentials, benefits and use values that well-functioning ecosystems provide to humans and the biosphere (e.g. Spangenberg et al. 2014) and as an end point (value to be protected) in ecological risk assessment of chemicals. Neonicotinoid insecticides and fipronil are frequently detected in environmental media (soil, water, air) at locations where no pest management benefit is provided or expected. Yet, these media provide essential resources to support biodiversity and are known to be threatened by long-term or repeated contamination. The literature synthesized in this integrated assessment demonstrates the large-scale bioavailability of these insecticides in the global environment at levels that are known to cause lethal and sublethal effects on a wide range of terrestrial (including soil) and aquatic microorganisms, invertebrates and vertebrates. Population-level impacts have been demonstrated to be likely at observed environmental concentrations in the field for insect pollinators, soil invertebrates and aquatic invertebrates. There is a growing body of evidence that these effects pose risks to ecosystem functioning, resilience and the services and functions provided by terrestrial and aquatic ecosystems. Such services and functions can be provisioning, regulating, cultural or supporting and include amongst others soil formation, soil quality, nutrient cycling, waste treatment and remediation, pollination, food web support, water purification, pest and disease regulation, seed dispersal, herbivory and weed control, food provision (including fish), aesthetics and recreation.

## Knowledge gaps

While this assessment is based on a growing body of published evidence, some knowledge gaps remain. These compounds have been subject to regulatory safety tests in a number of countries. However, several potential risks associated with the present global scale of use are still poorly understood. We highlight key knowledge gaps.

- For most countries, there are few or no publicly available data sources on the quantities of systemic pesticides being applied, nor on the locations where these are being applied. Reliable data on the amounts used are a necessary condition for realistic assessments of ecological impacts and risks.
- Screening of neonicotinoid and fipronil residues in environmental media (soils, water, crop tissues, non-target vegetation, sediments, riparian plants, coastal waters and sediments) is extremely limited. Although their water solubility and propensity for movement are known, also, only very scarce data for marine systems exist.
- An even bigger knowledge gap is the environmental fate of a wide range of ecotoxic and persistent metabolites of neonicotinoids and fipronil. Hence, we cannot evaluate with accuracy the likely joint exposure of the vast majority of organisms.
- There is a poor understanding of the environmental fate of these compounds, and how, for example, soil properties affect persistence and whether they accumulate in (usually flowering) woody plants following repeated treatments with the parent compound. The behaviour of degradation products (which can be highly toxic and persistent) in different media (plants, soils, sediments, water, food chains, etc.) is poorly known.
- Long-term toxicity to most susceptible organisms has not been investigated. For instance, toxicity tests have only been carried out on four of the approximately 25,000 globally known species of bees, and there are very few studies of toxicity to other pollinator groups such as hoverflies or butterflies and moths. Similarly, soil organisms (beyond earthworms) have received little attention. Soil organisms play multiple roles in the formation of soil and in the maintenance of soil fertility. Toxicity to vertebrates (such as granivorous mammals and birds which are likely to consume treated seeds) has only been examined in a handful of species.
- Those toxicological studies that have been performed are predominantly focused on acute toxicity tests, whereas the effects of long-term, acute and chronic exposure is less well known, despite being the most environmentally relevant scenario for all organisms in agricultural and aquatic environments. The long-term consequences of exposure under environmentally realistic conditions have not been studied.
- All neonicotinoids bind to the same nAChRs in the nervous system such that cumulative toxicity is expected. At present, no studies have addressed the additive or synergistic effects of simultaneous exposure to multiple compounds of the neonicotinoid family, i.e. imidacloprid, clothianidin, thiamethoxam, dinotefuran, thiacloprid, acetamiprid, sulfoxaflor, nitenpyram, imidaclothiz, paichongding and cycloxaprid, into an aggregated dose of e.g. “imidacloprid equivalents”. Currently, risk assessments are done for each chemical separately, while many non-target species, such as pollinators, are simultaneously being exposed to multiple neonicotinoids as well as other pesticides and stressors. As a consequence, the risks have been systematically underestimated. While quantifying the suite of co-occurring pesticides is largely an intractable problem, a single metric that incorporates all neonicotinoid exposures to representative taxa would be an invaluable starting point.
- Cumulative toxicity of successive and simultaneous exposure has not been studied in the regulatory assessment and governance of chemical risks.
- Sublethal effects that often have lethal consequences in a realistic environmental setting have not been studied in most organisms. However, they are known to be profound in bees, and for those few other species where studies have been performed, sublethal doses of these neurotoxic chemicals have been reported to have adverse impacts on behaviour at doses well below those that cause immediate death.
- Interactions between systemic insecticides and other stressors, such as other pesticides, disease and food stress, have been explored in only a handful of studies (on bees), and these studies have revealed important synergistic effects. For example, in honeybees, low doses of neonicotinoids greatly increase susceptibility to viral diseases. Interactions between systemic insecticides and other stressors in organisms other than bees are almost entirely unstudied. In field situations, organisms will almost invariably be simultaneously exposed to multiple pesticides as well as other stressors, so our failure to understand the consequences of these interactions (or even to devise suitable means to conduct future studies in this area) is a major knowledge gap.
- Impacts of these systemic insecticides on the delivery of a wide range of ecosystem services are still uncertain. The accumulation in soil and sediments might lead us to predict impacts on soil fauna such as earthworms and springtails (Collembola), which may in turn have consequences for soil health, soil structure and permeability and nutrient cycling. Contamination of field margin vegetation via dust or ground or surface water might lead us to expect impacts on fauna valued for aesthetic reasons (e.g. butterflies) and is likely to impact populations of important beneficial insects that deliver pollination or pest control services (e.g. hoverflies, predatory beetles). The general depletion of farmland and aquatic insect populations is likely to impact insectivorous species such as birds and bats. Contamination of freshwater is hypothesized to reduce invertebrate food for fish and so impact fisheries. The same might apply to coastal marine systems, potentially posing serious threats to coral reefs and salt marsh estuaries. None of these scenarios have been investigated.
- The short- and long-term agronomic benefits provided by neonicotinoids and fipronil are unclear. Given their use rates, the low number of published studies evaluating their benefit for yield or their cost-effectiveness is striking, and some recent studies (see Furlan and Kreutzweiser 2014) suggest that their use provides no net gain or even a net economic loss on some crops. It is not currently known what the impact on farming would be if these systemic pesticides were not applied or applied less (though their recent partial withdrawal in the EU provides an opportunity for this to be examined).

Given these knowledge gaps, it is impossible to properly evaluate the full extent of risks associated with the ongoing use of systemic insecticides, but the evidence reviewed in this special issue suggests that while the risks affect many taxa, the benefits have not been clearly demonstrated in the cropping systems where these compounds are most intensively used.

## Conclusions

Overall, the existing literature clearly shows that present-day levels of pollution with neonicotinoids and fipronil caused by authorized uses (i.e. following label rates and applying compounds as intended) frequently exceed the lowest observed adverse effect concentrations for a wide range of non-target species and are thus likely to have a wide range of negative biological and ecological impacts. The combination of prophylactic use, persistence, mobility, systemic properties and chronic toxicity is predicted to result in substantial impacts on biodiversity and ecosystem functioning. The body of evidence reviewed in this Worldwide Integrated Assessment indicates that the present scale of use of neonicotinoids and fipronil is not a sustainable pest management approach and compromises the actions of numerous stakeholders in maintaining and supporting biodiversity and subsequently the ecological functions and services the diverse organisms perform.

In modern agricultural settings, it is increasingly clear that insecticide treatments with neonicotinoids and fipronil—and most prominently its prophylactic applications—are incompatible with the original mindset that led to the development of the principles of integrated pest management (IPM). Although IPM approaches have always included insecticide tools, there are other approaches that can be effectively incorporated with IPM giving chemicals the position of the last resort in the chain of preferred options that need be applied first. Note that the current practice of seed treatment is the opposite: it applies chemicals as the first applied option instead of the last resort. The preferred options include organic farming, diversifying and altering crops and their rotations, inter-row planting, planting timing, tillage and irrigation, using less sensitive crop species in infested areas, using trap crops, applying biological control agents, and selective use of alternative reduced-risk insecticides. Because of the persistent and systemic nature of fipronil and neonicotinoids (and the legacy effects and environmental loading that come with these properties), these compounds are incompatible with IPM. We accept that IPM approaches are imperfect and constantly being refined. However, there is a rich knowledge base and history of success stories to work from in many systems where pest management is required. In fact, in Europe, the IPM approach has become compulsory for all crops as of the 1st of January 2014 in accordance with EU Directive 2009/128/EC, but most member states still need to operationalize and implement this new regulation, and IPM is sometimes poorly defined.

## Recommendations

The authors suggest that regulatory agencies consider applying the principles of prevention and precaution to further tighten regulations on neonicotinoids and fipronil and consider formulating plans for a substantial reduction of the global scale of use. Continued research into alternatives is warranted, but equally pressing is the need for education for farmers and other practitioners and the need for policies and regulations to encourage the adoption of alternate agricultural strategies to manage pests (e.g. IPM, organic, etc.). In addition, there is a need for research to obtain a better understanding of the institutional and other barriers that hamper large-scale adoption of proven sustainable agricultural practices that can serve as alternatives to the use of neonicotinoids and fipronil—as of many other pesticides as well.

The adequacy of the regulatory process in multiple countries for pesticide approval must be closely considered and be cognizant of past errors. For example, other organochloride insecticides such as DDT were used all over the world before their persistence, bioaccumulation and disruptive impacts on ecosystem functioning were recognized, and they were subsequently banned in most countries. Organophosphates have been largely withdrawn because of belated realization that they posed great risks to human and wildlife health. The systemic insecticides, neonicotinoids and fipronil, represent a new chapter in the apparent shortcomings of the regulatory pesticide review and approval process that do not fully consider the risks posed by large-scale applications of broad-spectrum insecticides.
